# On the Internal Use of Stramonium

**Published:** 1817-04

**Authors:** Henry Ward


*For the London Medical and Physical Journal.*

### *On the internal Use of Stramonium;* by Henry Ward, Esq.

THE fashionable practice of quackery (if I may so call it) is become so notorious as to be disgusting to every scientific practitioner. Monopoly in physic is disgraceful, and it is much to be regretted, that patents or mock degrees should be imposed on the public in the manner we are daily seeing, to the discredit of medicine, as well as the ruin of so many constitutions. For this reason, the exertions of regular practitioners in communicating medical facts, through the channel of respectable publications, both abroad and at home, is become more essential to the advancement of professional knowledge, and for the benefit of mankind. With this view, I beg leave to offer the result of several diseases peculiar to the lungs and chest, which have yielded to a preparation of the stramonium, given in doses according to the nature of the complaint and condition of the patient.

Having for a length of time witnessed the good effects of the Datura stramonium, in asthmatic and catarrhal affections, when smoaked in lieu of tobacco, I was induced to prepare a tincture after the following formula.

℞. Stramonii

℞. Stramonii Exsic. et Incisi. f℥iv. Spt. Vini Tenuis, f℥xv. Spt. Ammon. c. f℥j. Macera dies per xiv. et Cola.

The above is not offered as a never failing remedy, (for in medicine I know of no such thing,) but, in the generality of cases where it has been given, it has been with the most pointed success. The dose is from ♏xiv. ad ♏xxiv. gradually increased. One patient took fʒſs. which produced a slight nausea, but not the least degree of vertigo, as was imputed to this plant by several writers. In many cases, it has, I think, succeeded better than the squill. In cases where the chest has been greatly troubled with phlegm, an emetic of Pulv. Ipecac. has succeeded its use.

To relate a number cases would be to say no more than I have already related. All the patients to whom I have given the tincture have been labouring under asthmatic and catarrhal affections; but the generality of them have found the breathing extremely assisted by it. This, I trust, will be sufficient to induce your readers to give the remedy a fair trial, and communicate the result.

*Sloane-street; March 20, 1817.*

---

*For the London Medical and Physical Journal.*

